# The impact of bipolar disorder on the parent–infant relationship

**DOI:** 10.1192/j.eurpsy.2026.10284

**Published:** 2026-07-31

**Authors:** A. Biaggi, K. Hazelgrove, P. Dazzan, Psychiatry Research and Motherhood study (PRAM)

**Affiliations:** 1 Pharmacological and Biomolecular Sciences, University of Milan, Milan, Italy; 2 Psychological Medicine, King’s College London; 3 National Institute for Health Research (NIHR) Mental Health Biomedical Research Centre at South London and Maudsley NHS Foundation Trust and King’s College London, London, United Kingdom

## Abstract

**Introduction:**

Very little research to date has been conducted to investigate mother-infant relationship and infant development in women with a history of bipolar disorder or affective psychosis.

*Objectives*: The current study (Biaggi et al. J Affect Disord 2021; Biaggi et al. Psychol Med 2023; Biaggi et al. J Affect Disord 2024) aimed to investigate the quality of the mother-infant relationship and the development of the infants born to women with a history of bipolar disorder, schizoaffective disorder or previous postpartum psychosis (PP). Potential predictors of mother-infant interaction quality were also investigated.

**Methods:**

103 women (and their offspring) were included in the study, 43 with a history of bipolar disorder, schizoaffective disorder or previous postpartum psychosis, and 60 with no current/previous mental illness or family history of PP. Women were recruited during pregnancy and followed up with their offspring until 12 months postpartum.

Mother–infant relationship was assessed using the MAAS in pregnancy, the MPAS and the CARE-Index at 8 weeks and 12 months postpartum. Infant development was investigated using the Bayley-III at 12 months postpartum.

**Results:**

Compared to healthy controls*,* women with a history of bipolar disorder or affective psychosis had less synchronous mother–infant interactions, a more negative affective experience towards their infants at both 8 weeks and 12 months postpartum and had infants with less optimal cognitive, language, motor and socio-emotional development at 12 months postpartum. Maternal ability to recognize emotions, particularly fear and infant social-interactive behaviour were significant predictors for mother-infant interaction quality.

**Conclusions:**

These results suggest that a history of bipolar disorder or affective psychosis could represent a risk factor for the mother-infant relationship and the infant development during the perinatal period, suggesting the need of early monitoring and the development of interventions, where necessary. Preventive interventions aimed at improving maternal ability to recognize emotions and infant social-interactive behaviour may be helpful to support the quality of the mother-infant interaction and potentially also the infant long-term development.

**Disclosure of Interest:**

None Declared

